# Etymologia: malaria

**DOI:** 10.3201/eid1207.ET1207

**Published:** 2006-07

**Keywords:** Etymologia, malaria

## [mə-lar′e-ə]

*Malaria*, “bad air” in Italian, was blamed for the deaths of >1,000 workers digging the Erie Canal in 1819. Work on the canal continued in winter, when the swamp was frozen over (and, although the vector was not known at the time, mosquitoes were dormant). Malaria, caused by parasites of the genus *Plasmodium* and usually transmitted by the bite of infected *Anopheles* mosquitoes, is endemic in many warm regions. Charles Louis Alphonse Laveran discovered the protozoan cause of malaria in 1880. The Office of Malaria Control in War Areas, which was established in 1942 to control malaria and other vectorborne diseases in the southern United States, evolved into what is today the Centers for Disease Control and Prevention.

Sources: Dorland’s illustrated medical dictionary. 30th ed. Philadelphia: Saunders; 2003; cdc.gov; and wikipedia.org

